# Hybrid Feature Learning for Wearable Stress Detection: Combining Domain Knowledge with Supervised Deep Learning

**DOI:** 10.3390/s26113451

**Published:** 2026-05-29

**Authors:** Dennis Birkenmaier, Shanthan Rao Kanuganti, Wilhelm Stork

**Affiliations:** 1 FZI Research Center for Information Technology, 76131 Karlsruhe, Germany; kanuganti@fzi.de; 2 Institute for Information Processing Technologies, Karlsruhe Institute of Technology KIT, 76131 Karlsruhe, Germany; wilhelm.stork@kit.edu

**Keywords:** electrodermal activity, stress detection, wearable sensors, feature extraction, deep learning, supervised learning, cvxEDA decomposition, artifact detection, WESAD dataset

## Abstract

Accurate stress monitoring is critical for high-risk professions like firefighting, yet existing wearable solutions face challenges balancing accuracy with practical usability. While electrodermal activity (EDA) offers a non-invasive, single-sensor approach, current automated feature extraction methods fail to capture stress-discriminative patterns effectively. We developed a hybrid stress detection pipeline combining 20 hand-crafted physiological features with 32 deep-learned features from a supervised convolutional autoencoder. Unlike traditional unsupervised approaches optimized solely for signal reconstruction, our architecture employs a dual-head design with weighted classification loss to guide feature learning toward stress discrimination. The system was validated on the WESAD dataset (15 subjects) using rigorous leave-one-subject-out (LOSO) cross-validation, along with comprehensive preprocessing, including cvxEDA decomposition, adaptive artifact detection, and physiological peak validation. Our optimized K-Nearest Neighbors classifier achieved 98.62% accuracy, surpassing the industry-standard PyEDA benchmark (97.0%) by 1.62 percentage points. The model demonstrated 97.58% sensitivity (true positive rate) and 98.92% specificity (true negative rate), with only 2.42% false negatives—critical for safety-critical applications. Ablation studies revealed that unsupervised autoencoder features alone achieved only 55% accuracy, increasing to 89% with supervised learning and 98.62% with the hybrid approach, representing a 43.62-percentage-point improvement. This work demonstrates that combining domain-specific physiological knowledge with label-aware deep learning produces more discriminative features than either approach alone. The resulting system successfully translates complex probabilistic outputs into an interpretable 1–10 stress score, providing a practical foundation for real-time stress monitoring in wearable devices for first responders.

## 1. Introduction

High-risk professions, such as firefighting, operate under an elevated risk profile where psychological stress can have severe consequences. The significance of stress evaluation in these fields is critical, yet existing wearable solutions often face challenges in balancing accuracy with practical usability. While physiological monitoring offers a pathway to mitigate these risks, current automated feature extraction methods frequently fail to capture stress-discriminative patterns effectively.

Electrodermal Activity (EDA) presents a viable non-invasive, single-sensor approach for monitoring emotional arousal. Based on Ohm’s law, EDA measures small, involuntary changes in the skin’s electrical properties caused by microscopic moisture activated within the sweat glands via the Sympathetic Nervous System (SNS). Its operational efficiency, characterized by low-cost hardware and low power consumption, makes it the best platform for creating large-scale, reliable systems for monitoring physiological activity. However, the measurement is highly sensitive to environmental factors, such as heat or humidity, which can naturally alter skin conductance. Additionally, sensor and electrode issues, along with electrical interference, can introduce artifacts that obscure the true stress signal.

Research in EDA-based stress detection has evolved considerably in recent years, with approaches ranging from rule-based algorithms to machine learning pipelines. Kyriakou et al. proposed a rule-based algorithm combining galvanic skin response and skin temperature measurements from the Empatica E4 wristband to detect moments of stress, achieving 84% accuracy in real-world field studies validated through a mixed-methods approach including ego-perspective videos and questionnaires [1]. While their approach demonstrated transferability from laboratory to real-world settings, accuracy remained limited due to the reliance on predefined rules and the use of only two physiological signals. Zhu et al. conducted a systematic evaluation of five machine learning classifiers on wrist-based EDA signals across four public datasets, reporting a peak accuracy of 92.9% with SVM on the VerBIO dataset and 86.5% with Random Forest on WESAD [2]. Their findings confirmed that EDA outperforms other wrist-available modalities (PPG, ECG) for stress classification and that female subjects exhibited higher classification accuracy than males, but their approach relied on conventional statistical features without leveraging deep learning-based feature extraction. Hosseini et al. demonstrated that careful feature selection and per-subject normalization can substantially improve EDA-based stress detection, achieving 97.03% accuracy on the WESAD dataset using only five selected statistical features from the Empatica E4 wrist sensor with an AdaBoost classifier [3,4]. Their work highlighted the importance of addressing inter-individual variability through normalization but was limited to wrist-sensor data, which is inherently more susceptible to motion artifacts than chest-placed EDA. Across these studies, a common limitation persists: approaches relying solely on hand-crafted statistical features cannot capture complex non-linear signal dynamics, while purely data-driven deep learning methods often lack physiological interpretability and require substantial computational resources unsuitable for embedded wearable platforms.

To address these limitations, this work proposes a hybrid stress detection pipeline. The development of the model was not a one-step success but rather an evolving development resulting from a root-cause analysis of failures in an iterative testing approach. Initial unsupervised approaches achieved only 55% accuracy, focusing purely on reconstruction. By integrating supervised learning and hybrid features, performance increased significantly, culminating in optimized classifiers achieving 98.62% accuracy. This represents a total improvement of 43.62 percentage points over the baseline.

This paper is structured as follows: First, we describe the dataset and sensor platform used for data collection, along with the experimental protocol and ground truth labeling. Next, we detail the signal processing pipeline, including filtering, downsampling, and artifact detection and removal strategies. We then present the feature extraction methods, including both hand-crafted physiological features and deep-learned features from a supervised convolutional autoencoder. Finally, we evaluate the performance of our hybrid model against industry benchmarks and discuss the implications of our findings for real-time stress monitoring in wearable devices for first responders.

## 2. Materials and Methods

### 2.1. Dataset and Sensor Platform

This study utilized the publicly available WESAD (Wearable Stress and Affect Detection) dataset [5]. The dataset comprises physiological recordings from 15 participants (from an initial cohort of 17; two were excluded due to technical issues). Data were collected using two wearable sensor platforms simultaneously, each representing a different form factor and placement strategy for physiological monitoring.

The RespiBAN Professional chest strap is a research-grade, multi-modal physiological sensor platform. In the WESAD study, it recorded six signal modalities: electrocardiography (ECG), electrodermal activity (EDA), electromyography (EMG), three-axis accelerometry (ACC), skin temperature, and respiration rate. All channels were sampled synchronously at 700 Hz, providing high temporal resolution suitable for capturing rapid physiological transients. The EDA channel of the RespiBAN employs the exosomatic (constant-voltage) measurement method, in which a low-amplitude direct-current (DC) voltage is applied across two Ag/AgCl electrodes placed on the chest. Changes in skin conductance, expressed in microsiemens (μS), are measured as variations in the resulting current flow. Because the chest placement ensures stable electrode–skin contact during body movement and minimizes the mechanical displacement artifacts common at distal sites (e.g., wrist, fingers), the chest EDA channel provides a substantially cleaner signal than wrist-based alternatives.

The second platform was the Empatica E4 wristband, a research-grade wearable worn on the participants’ non-dominant hand [5]. In contrast to the RespiBAN’s synchronous 700 Hz acquisition, the E4 records modality-specific sampling rates: blood volume pulse (BVP) at 64 Hz, three-axis accelerometry at 32 Hz, and both electrodermal activity and skin temperature at 4 Hz. The EDA channel uses two silver-coated stainless-steel electrodes (8 mm diameter) on the wrist volar surface, providing a measurement resolution of 900 pS over an operating range of 0.01–100 μS [6]. While this wrist-based form factor offers substantially higher long-term wearability than a chest strap, the 4 Hz sampling rate constrains the spectral resolution of phasic SCR analysis, and the distal placement renders the signal considerably more susceptible to motion artifacts from hand and arm movement [7]. For these reasons, the chest-placed RespiBAN EDA channel was selected as the primary signal source in this study, while the E4 placement is retained as a reference for the discussion of field-deployment limitations (Section 4.5).

Although multi-modal sensor fusion (combining EDA with ECG, EMG, ACC, and other modalities) has been shown to improve stress detection accuracy, it requires complex sensor setups involving chest straps, adhesive electrodes, and multiple devices that are impractical for real-world deployment in high-risk occupations such as firefighting. EDA was selected as the sole sensing modality for this study due to three sensor-specific advantages. First, EDA provides high specificity for sympathetic nervous system (SNS) activation: the eccrine sweat glands responsible for EDA variations are innervated exclusively by the SNS, unlike cardiovascular measures (ECG, BVP) that reflect both SNS and parasympathetic nervous system (PNS) activity. Second, EDA is an involuntary response that cannot be consciously suppressed by the user, ensuring measurement integrity even in high-stress scenarios. Third, the hardware requirements for EDA sensing are minimal—two electrodes and a low-power constant-voltage circuit—making it the most practical modality for integration into lightweight, low-cost wearable form factors such as wristbands or smartwatches.

#### Electrodermal Activity

Electrodermal activity (EDA), also referred to as galvanic skin response (GSR) or skin conductance, quantifies changes in the electrical conductance of the skin caused by variations in eccrine sweat gland activity [8,9]. The measurement follows Ohm’s law: a constant low-amplitude DC voltage is applied between two electrodes in contact with the skin, and the resulting current is measured [10]. When the sympathetic nervous system (SNS) activates the sweat glands in response to a stressor, the sweat ducts fill with an ionic solution, thereby increasing the electrical conductivity of the stratum corneum. The sensor converts this increase in current flow into a conductance value expressed in microsiemens (μS). Importantly, even in cold environments where visible perspiration is absent, EDA sensors can detect the sub-threshold activation of sweat glands at a microscopic level, making the measurement sensitive to subtle autonomic changes [9].

The raw EDA signal comprises two physiologically distinct components that must be separated for accurate stress detection [8]. The tonic component, or skin conductance level (SCL), represents the slowly varying baseline conductance reflecting general autonomic arousal over minutes to hours. The phasic component, or skin conductance response (SCR), consists of rapid, transient peaks that occur within 1–5 s following a discrete stimulus or stressor [9]. SCR peaks are characterized by a fast rise phase and a slower exponential recovery phase [8]. For stress quantification, the phasic SCR component is of primary interest, as it captures event-related sympathetic activation, while the tonic SCL provides information about sustained arousal levels [10].

The sensor-specific challenges of EDA measurement that directly influenced the processing pipeline developed in this study include: (1) motion artifacts caused by electrode displacement during physical movement, which produce sharp signal transients indistinguishable in shape from genuine SCR peaks; (2) environmental sensitivity, as high ambient temperature and humidity can elevate the tonic SCL independently of psychological stress; (3) individual variability in baseline skin conductance due to differences in skin thickness, hydration, age, and SNS sensitivity, necessitating subject-specific processing; and (4) electrical noise from poor electrode contact or external electromagnetic interference.

The experimental protocol and ground truth annotations follow the original WESAD study design described by Schmidt et al. [5] and are summarized here for completeness. Participants were exposed to four clearly defined affective conditions: (1) Baseline—participants sat quietly reading neutral magazines; (2) Stress—induced via the Trier Social Stress Test (TSST), involving a public speaking task and a mental arithmetic task performed in front of an audience; (3) Amusement—elicited by viewing a standardized set of humorous video clips; and (4) Meditation—a guided relaxation phase following the Innere Stille protocol. Ground truth was provided by condition labels synchronized with the RespiBAN’s 700 Hz sampling clock (label codes: 0 = transition, 1 = baseline, 2 = stress, 3 = amusement, 4 = meditation) and by self-report questionnaires (PANAS, STAI, SAM) completed after each condition. For the binary classification task evaluated in this study, stress-label windows (code 2) were treated as the positive class and baseline-label windows (code 1) as the negative class; transition and amusement/meditation windows were excluded from the binary analysis. Subject S12 was excluded during preprocessing due to data quality issues documented in the original dataset release [5], resulting in a final sample of 14 subjects (S2–S17, excluding S12).

### 2.2. Signal Processing

#### 2.2.1. Filtering

**Figure 1:**
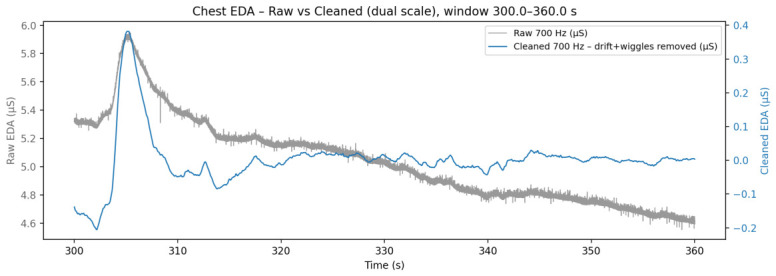
Exemplary comparison of raw and filtered EDA signals.

The raw EDA signal acquired by the RespiBAN chest sensor at 700 Hz was conditioned using two sequential fourth-order Butterworth filters to remove frequency components outside the physiologically relevant bandwidth. First, a high-pass filter with a cutoff frequency of 0.05 Hz was applied to eliminate baseline drift, a slow, non-physiological shift of the signal’s zero point caused by factors such as gradual changes in ambient temperature, slow variations in electrode–skin contact pressure, or warm-up of the sensor’s analog circuitry. The 0.05 Hz cutoff corresponds to the longest period (20 s) at which a genuine autonomic response can plausibly occur and has been established as the standard high-pass setting for separating phasic SCR activity from sub-physiological drift in modern EDA processing pipelines [10,11] that is also the default high-pass setting in widely used commercial analysis software [12]. Subsequently, a low-pass filter with a cutoff frequency of 5 Hz was applied. This cutoff served two purposes: noise reduction, as the minimum rise time of a genuine SCR peak is approximately 1 s, meaning that any signal content above 5 Hz represents electrical noise, high-frequency sensor jitter, or electromagnetic interference rather than physiological activity; and anti-aliasing, as the Nyquist criterion requires all frequency content above half the target sampling rate (10 Hz for a 20 Hz target) to be removed before downsampling to prevent spectral folding artifacts. The conservative 5 Hz cutoff provides an additional safety margin above the Nyquist limit. An exemplary comparison of the raw and filtered EDA signals is shown in Figure 1, demonstrating the effective removal of low-frequency drift and high-frequency noise while preserving the integrity of SCR peaks.

#### 2.2.2. Downsampling

**Figure 2:**
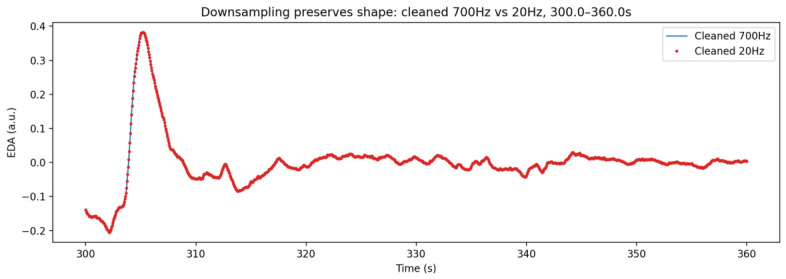
Exemplary comparison of original and downsampled EDA signals.

Following filtering, the signal was downsampled from the RespiBAN’s native 700 Hz to a computationally efficient rate of 20 Hz using the nearest-neighbor resampling method. This 35-fold reduction in data volume was justified by the temporal dynamics of the target signal: the fastest physiological event of interest—the SCR rise phase—occurs over a minimum of 300 ms, which at 20 Hz is still captured by at least 6 samples, providing sufficient temporal resolution to preserve peak morphology and kinetics. The integrity of the downsampled signal was verified through visual overlay of the original 700 Hz waveform and the 20 Hz resampled data points, confirming that no distortion of SCR peak shape occurred. An exemplary comparison of the raw and downsampled EDA signals is shown in Figure 2, illustrating that the downsampled signal retains all physiologically relevant features while significantly reducing data size for subsequent processing. A metric-wise comparison of the dataset before and after downsampling is provided in Table 1, demonstrating a substantial reduction in total samples and duration while maintaining an acceptable signal-to-noise ratio (SNR) and achieving a mean SNR improvement of +79.7% across all subjects while retaining 97.4% of recording duration. The slight increase in movement ratio (from 0.044 to 0.060) is attributable to the anti-aliasing filter attenuating high-amplitude transient spikes that coincidentally suppressed some short-duration movement events at 700 Hz; residual artifacts were addressed in the subsequent two-stage detection procedure.

### 2.3. Artifact Detection and Removal

A two-stage, physiologically informed artifact detection and correction procedure was developed to address the sensor-specific noise sources inherent in EDA measurement. Although the controlled laboratory setting of the WESAD study minimized extreme motion artifacts and environmental fluctuations compared to field conditions, the raw sensor signal still contained contamination from electrode micro-movements, brief electrical interference, and occasional sensor contact issues that required systematic identification and removal.

#### 2.3.1. Stage 1: Conservative Artifact Detection

Four categories of sensor artifacts were detected using adaptive thresholds based on the median absolute deviation (MAD) of the cleaned EDA signal (Threshold=Factor×MAD), allowing the detection sensitivity to scale automatically with each subject’s individual signal characteristics—a critical requirement given the high inter-subject variability in EDA baseline levels and signal dynamics. The use of MAD rather than the conventional standard-deviation-based criterion was motivated by its well-established robustness to the very outliers it is meant to detect [13]. The MAD was computed once per subject over the full recording length, following the practice adopted in prior EDA artifact-detection work for wearable sensors [14].

**Figure 3:**
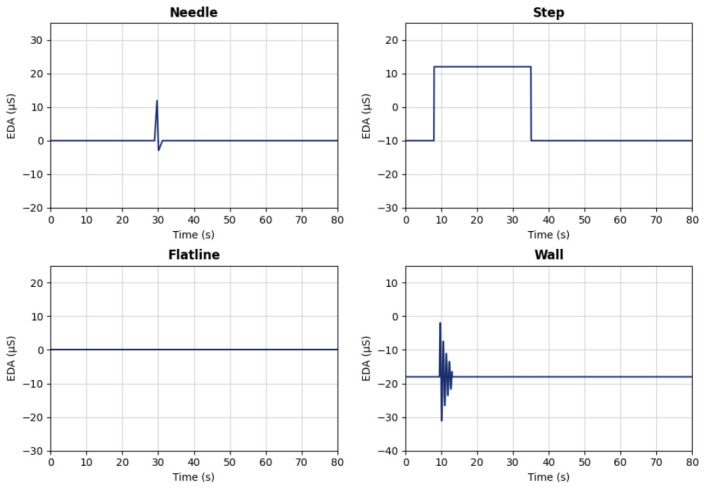
Examples for occurring artifact types.

The artifact categories directly correspond to the physical failure modes of the EDA sensor: (a) Step artifacts—large, abrupt, sustained signal shifts typically caused by mechanical displacement of the electrode against the skin (step factor =12.0); (b) Needle artifacts—sharp, brief spikes of one to two samples duration, resulting from momentary loss of electrode–skin contact or external electrical interference (needle factor =10.0), assessed via a local outlier score relative to the local MAD; (c) Flatline artifacts—segments with near-zero variability (rolling standard deviation <0.001 relative to overall signal standard deviation), indicating complete sensor disconnection or electronic malfunction; and (d) Wall artifacts —extremely abrupt jumps confined to one or two samples where more than 90% of the total amplitude change was concentrated in the top two consecutive samples, a pattern physically impossible for genuine physiological responses but characteristic of sudden sensor hardware events. The step and needle factors were selected empirically through visual inspection across all subjects to minimize false positives at the conservative detection stage; these values are intentionally well above the canonical 2.5–3MAD recommendation for outlier detection [13], accepting a higher initial flag rate that would subsequently be refined in the physiological validation stage (Section 2.3.2). Examples for each artifact type are shown in Figure 3.

#### 2.3.2. Stage 2: Physiological Validation

Each flagged segment was subjected to morphological validation against known SCR response characteristics to prevent false-positive artifact detection. This validation step is essential because genuine SCR peaks—particularly large-amplitude stress responses—can closely resemble step or needle artifacts in their raw waveform appearance. Biological timing rules were applied: a valid SCR must exhibit a rise time of 0.3–5.0 s and a recovery time of 1.0–20.0 s. The surrounding signal context (2 s before and 8 s after each candidate) was evaluated against these criteria. Flagged segments conforming to physiological SCR morphology were reclassified as valid responses. Across all subjects, 71.9% (1323 of 1841) of initially detected step artifacts were preserved as genuine SCR events through this validation mechanism, demonstrating the importance of physiological validation in preventing the loss of true stress-related signal content.

#### 2.3.3. Correction

**Figure 4:**
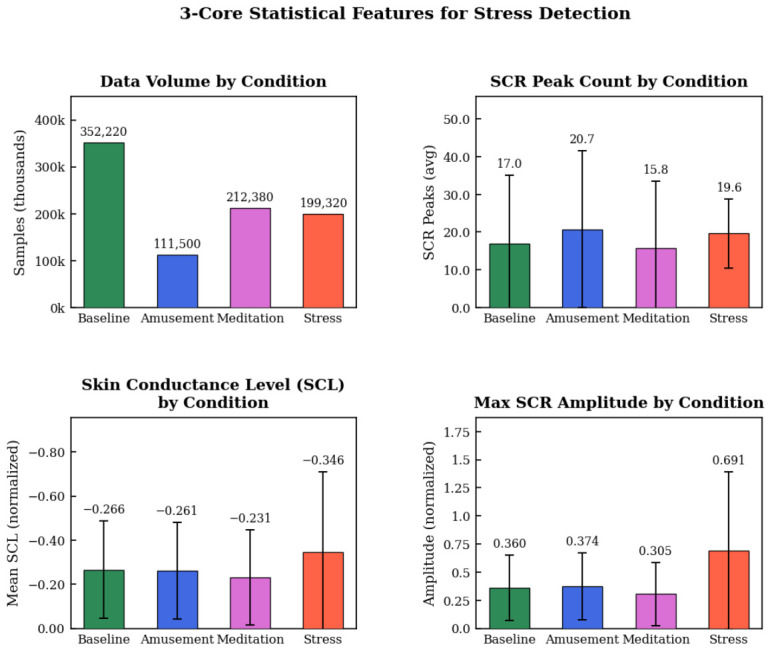
Overview of the artifact detection and correction process.

Remaining confirmed artifact segments less than 3.5 s in length were corrected via linear interpolation between clean anchor points, restoring signal continuity for downstream analysis. Following the two-stage detection and correction procedure, the residual uncorrected artifact rate across all subjects and experimental conditions was 0.0%: all 518 confirmed artifact segments (28.1% of the 1841 initially flagged candidates, after excluding the 1323 reclassified SCR events) were shorter than 3.5 s and were fully corrected via linear interpolation, retaining 100% of all time windows for subsequent analysis. This result is consistent with the controlled laboratory setting of the WESAD study and the mechanical stability of chest-worn EDA electrodes; it should not be interpreted as an absence of raw-signal noise, but rather as confirmation that the pipeline successfully resolved all detected contamination within the interpolation length limit. A brief overview of the artifact detection and correction process is shown in Figure 4, illustrating the initial conservative detection, physiological validation, and final corrected signal. The resulting clean EDA signal was then subjected to cvxEDA decomposition to isolate the tonic and phasic components for feature extraction.

Each flagged segment was subjected to morphological validation against known SCR response characteristics to prevent false-positive artifact detection. This validation step is essential because genuine SCR peaks—particularly large-amplitude stress responses—can closely resemble step or needle artifacts in their raw waveform appearance. Biological timing rules consistent with the canonical SCR morphology described in [8,9] were applied: a valid SCR must exhibit a rise time within 0.3–5.0s and a recovery time within 1.0–20.0s. These bounds are deliberately wider than the typical reference ranges (rise: 1–3s, half-recovery: 2–10s; [10]) to retain high-amplitude stress-related responses whose recovery dynamics are known to extend beyond those of low-arousal orienting responses. The surrounding signal context (2s before and 8s after each candidate) was evaluated against these criteria. Flagged segments conforming to physiological SCR morphology were reclassified as valid responses. Across all subjects, 71.9% (1323 of 1841) of initially detected step artifacts were preserved as genuine SCR events through this validation mechanism, demonstrating the importance of physiological validation in preventing the loss of true stress-related signal content.

### 2.4. Signal Decomposition Using cvxEDA

The cleaned EDA signal was decomposed into its tonic (SCL) and phasic (SCR) components using the cvxEDA algorithm [15]. This decomposition is critical for isolating the rapid, event-related SCR peaks from the slowly varying SCL baseline—a separation that cannot be achieved reliably with simple bandpass filtering alone due to spectral overlap between the two components. The cvxEDA method formulates the decomposition as a convex optimization problem according to the model ${\mathrm{EDA}}_{\mathrm{raw}}=\mathrm{SCL}+\mathrm{SCR}+\mathrm{Noise}$, subject to the constraints that the SCR component is sparse and positive (reflecting the physiology that sweat gland activation only increases conductance) and the SCL component is smooth. Since the original cvxEDA algorithm was designed and validated for data sampled at 250–1000 Hz using laboratory-grade EDA amplifiers, several parameter adaptations were required for the 20 Hz preprocessed signal from the RespiBAN sensor to avoid over-smoothing caused by the combined effect of the preprocessing low-pass filter and the internal smoothing of the cvxEDA algorithm. A comparison of cvxEDA parameters is provided in Table 2.

The rationale for each parameter adaptation is as follows.

Rise time constant $\tau_{0}$ (2.5s→2.0s). $\tau_{0}$ governs the onset speed of the modelled SCR impulse response. At 20 Hz the anti-aliasing low-pass filter (cut-off 9Hz) attenuates high-frequency transients, causing the apparent rise of individual SCRs to appear faster relative to the sampling grid. Reducing $\tau_{0}$ from 2.5s to 2.0s compensates for this effect and prevents the optimizer from over-attributing fast onsets to the noise term.

Recovery time constant $\tau_{1}$ (0.7s→1.0s). $\tau_{1}$ controls the decay of the SCR impulse response. Because downsampling to 20 Hz reduces the temporal resolution of the recovery phase, the phasic component appears to decay more slowly when reconstructed at coarser resolution. Increasing $\tau_{1}$ from 0.7s to 1.0s aligns the modelled decay envelope with the observable recovery dynamics at 20 Hz and avoids leakage of slow-decaying SCR tails into the tonic SCL estimate.

Sparsity regularization α ($8\times 10^{-4}\to 4\times 10^{-4}$). α penalizes the $\ell_{1}$-norm of the phasic driver signal, promoting sparse SCR activations. At 250–1000 Hz, where individual sample differences are small, strong sparsity is appropriate to suppress noise. At 20 Hz each sample represents a 50 ms interval, so physiologically valid SCR events that occupy only a few samples would be suppressed by the original $\alpha =8\times 10^{-4}$. Halving the penalty to $4\times 10^{-4}$ preserves low-amplitude but genuine SCR responses that would otherwise be zeroed out by over-regularization.

Tonic knot spacing $\delta_{\mathrm{knot}}$ (10s→20s). $\delta_{\mathrm{knot}}$ sets the spacing of the cubic-spline knots used to model the slowly varying SCL baseline. Denser knots (10 s) can track genuine SCL drift at high sampling rates without risk of overfitting, because the large number of samples per interval constrains the spline. At 20 Hz, a 10 s knot spacing yields only 200 samples per interval, making the spline susceptible to absorbing phasic SCR energy into the tonic baseline and thereby reducing SCR peak amplitude estimates. Doubling the knot spacing to 20 s enforces a smoother SCL trajectory and ensures that rapid conductance changes are assigned to the phasic component.

Peak detection threshold (fixed 0.05μS→ adaptive, condition-aware). The original fixed threshold of 0.05μS was established for high-resolution laboratory amplifiers with minimal baseline noise. At 20 Hz, tonic drift and residual anti-aliasing filter ringing produce a higher noise floor that would cause the fixed threshold to miss genuine low-amplitude SCRs in low-arousal conditions (e.g., baseline, meditation) while accepting noise peaks in high-arousal segments. The adaptive threshold scales with the rolling standard deviation of the phasic signal within each 60-s window and is conditioned on the experimental label (stress vs. non-stress), matching the psychophysiological expectation that SCR amplitude is substantially higher during stress (0.691±0.697μS) than during baseline (0.360±0.289μS).

### 2.5. Feature Extraction

#### 2.5.1. Statistical Feature Extraction

The continuous EDA signal was segmented into 60-s windows with 50% overlap (i.e., 30s step size), yielding 1200 samples per window at the 20 Hz sensor sampling rate. The 60-s window length was selected following the meta-analytic recommendation of Kreibig [16], who reports that response measures in autonomic emotion research are most commonly averaged over 30- or 60-s intervals; the same window length was adopted in the original WESAD baseline analysis [5], ensuring direct comparability with the established benchmark on this dataset. Only windows with ≥80% label purity (i.e., at least 80% of samples sharing the same experimental condition) were retained for analysis. This purity threshold was determined empirically as a balance between label fidelity at condition transitions and overall sample retention, resulting in 1374 windows (569 baseline, 317 stress, 169 amusement, 319 meditation).

**Figure 5:**
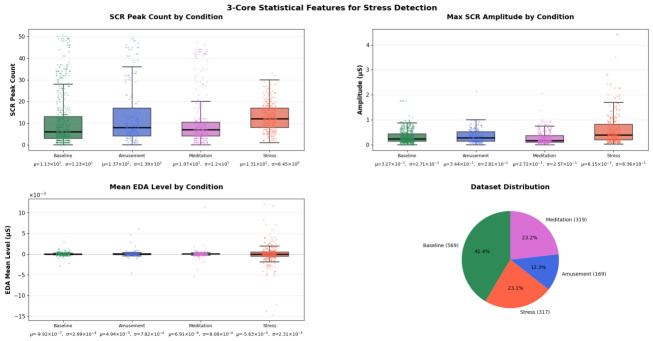
Overview of relevant feature behaviour according to experimental conditions.

Three primary statistical features were extracted per window from the decomposed SCR signal: (1) SCR peak count, using thresholds adapted from the psychophysiological literature to the sensor platform—a minimum amplitude prominence of 0.01μS, consistent with the established lower bound for distinguishable SCRs reported in [10]; a minimum inter-peak distance of 1s (corresponding to 20 samples at the operating rate of 20 Hz) to enforce temporal separability of overlapping responses [8]; and a minimum local rise of 0.005μS above the local baseline, deliberately set below the 0.01μS amplitude threshold to compensate for the additional smoothing introduced by the anti-aliasing filter prior to downsampling; (2) maximum SCR amplitude, defined as the height of the tallest SCR peak within the window, providing a direct measure of peak sympathetic activation intensity as captured by the sensor; and (3) mean EDA level across the window, reflecting the average tonic skin conductance. The results are shown in Figure 5, demonstrating that all three features exhibit significant differences between stress and baseline conditions, with higher peak counts, greater maximum amplitudes, and elevated mean levels during stress windows, consistent with the expected physiological response to acute stressors.

#### 2.5.2. Automatic Feature Extraction

**Figure 6:**
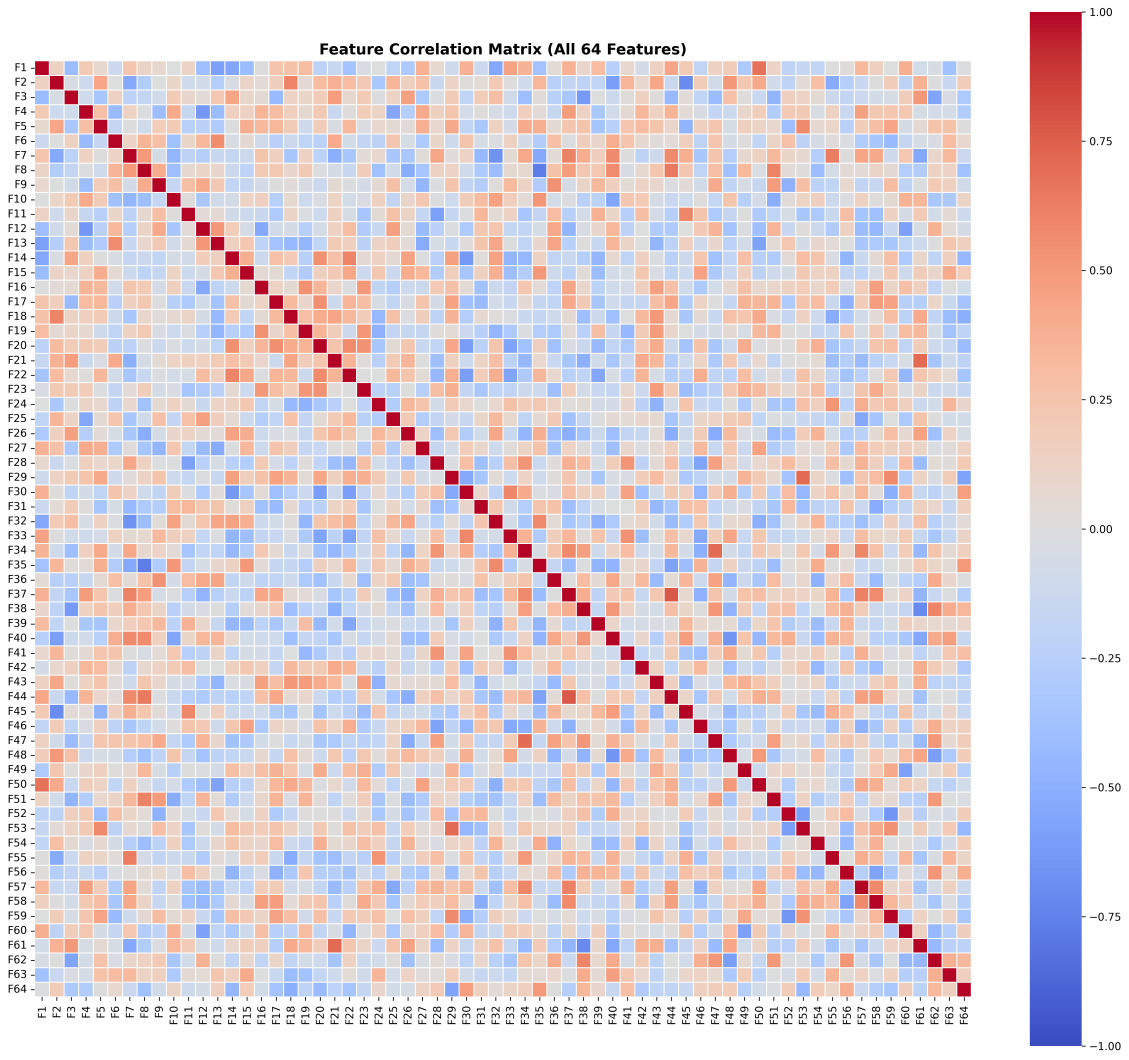
Automatic feature correlation matrix.

**Figure 7:**
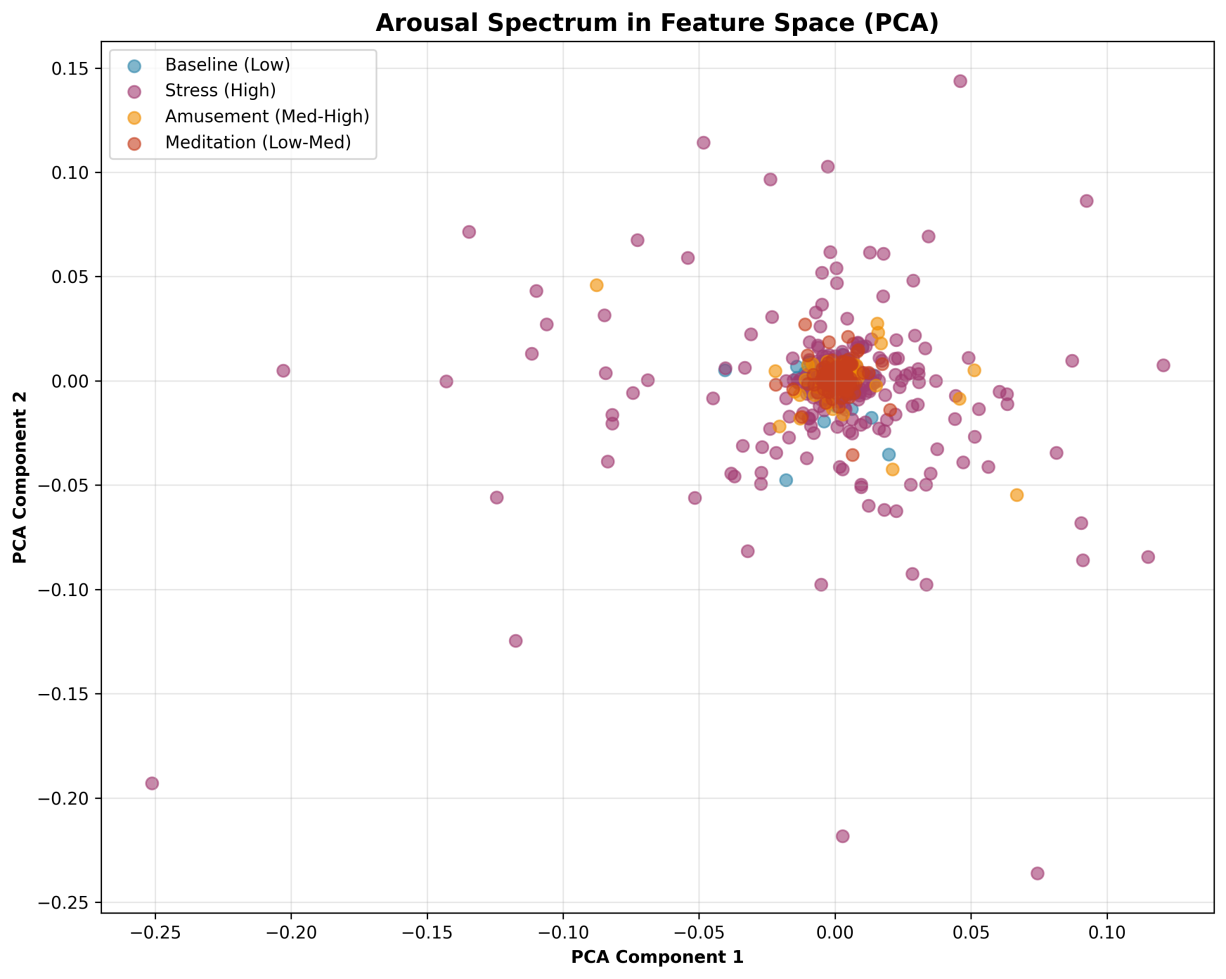
Automatic feature distribution across experimental conditions.

A custom deep convolutional autoencoder was developed for automatic feature learning from the raw sensor time series. Each 60-s window was zero-padded to 2048 samples for architectural compatibility with the convolutional layers. The encoder consisted of convolutional and pooling layers that progressively compressed each input window into a bottleneck of 64 latent features. The decoder mirrored this architecture using upsampling and convolution layers to reconstruct the original sensor signal. The autoencoder was trained using a compound loss function composed of four components: (1) Reconstruction error (MSE) to ensure information preservation; (2) a Feature diversity penalty to encourage non-redundant latent representations; (3) an Anti-saturation penalty to prevent features from saturating at extreme values; and (4) an Arousal ordering penalty to arrange the latent space such that windows representing different arousal levels (stress vs. baseline) are separated. The reconstruction quality was validated by overlaying original and reconstructed sensor signals, yielding MSE values of approximately 0.00009. Feature diversity was confirmed via pairwise Pearson correlation analysis of the 64 latent dimensions, with most off-diagonal values near zero. Arousal ordering was verified through PCA projection of the latent space, which showed clear separation between stress and baseline clusters. Figure 6 shows the feature correlation matrix, confirming low redundancy among the learned features, while Figure 7 illustrates the distribution of the latent features across experimental conditions, demonstrating that certain features are more active during stress windows compared to baseline, indicating successful learning of stress-discriminative patterns.

#### 2.5.3. Hybrid Feature Extraction

To overcome the limited classification performance of either individual approach, a hybrid feature extraction method was developed that combines domain-specific knowledge of EDA sensor physiology with data-driven deep learning. This approach fuses 20 hand-crafted statistical features with 32 latent features from a supervised autoencoder into a single 52-dimensional feature vector. The number of hand-crafted features (20) is determined directly by the physiologically motivated feature set defined in Table 3; no dimensionality selection was applied, as each feature encodes a distinct and interpretable aspect of the EDA signal (phasic activity, tonic level, signal quality, spectral content). The latent dimension of 32 was chosen by halving the unsupervised autoencoder’s bottleneck of 64, motivated by the observation that the supervised training objective already constrains the latent space toward a lower-dimensional, stress-discriminative manifold; retaining all 64 dimensions introduced redundancy without classification benefit, as confirmed by the feature correlation analysis shown in Figure 6. Both dimensionality choices were fixed prior to classifier training and LOSO evaluation, ensuring that no test-subject data influenced the feature representation design. The statistical features included, for the phasic (SCR) component: peak count per minute, average rise time, area under the curve, and maximum amplitude; and for the tonic (SCL) component: slope, variation, and overall mean. The full list of hand-crafted features is provided in Table 3. Additionally, signal-to-noise ratio—a metric directly reflecting the quality of the sensor output—and spectral energy in the 0–0.5 Hz band were computed.

**Figure 8:**
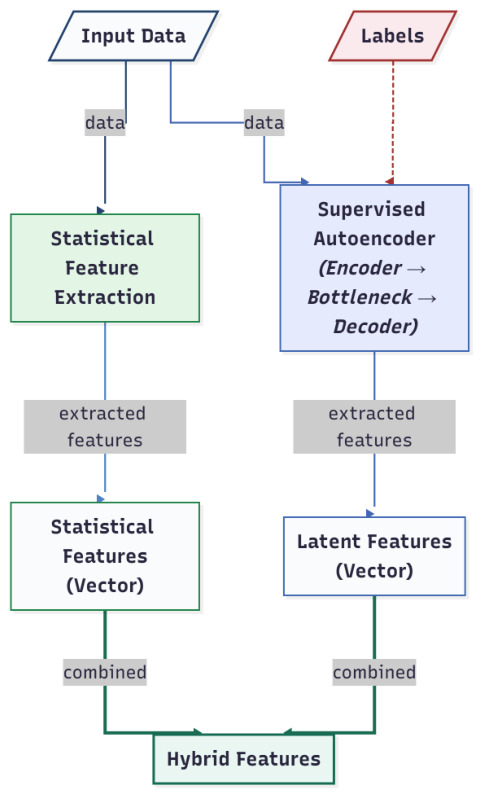
Hybrid feature extraction architecture.

The supervised autoencoder differed from the unsupervised version by employing a dual-head architecture with a compound loss function:

(1) $$L_{\mathrm{total}}=L_{\mathrm{reconstruction}}+\lambda \cdot L_{\mathrm{classification}}$$

where λ controls the relative weight of the classification loss (cross-entropy) versus the reconstruction loss (MSE). The value λ=3.0 was selected via a grid search over λ∈{0.5,1.0,2.0,3.0,5.0} using five-fold cross-validation on the training data only, with no access to the held-out LOSO test subjects at any stage of this search. The chosen value produced the highest mean validation accuracy while preserving acceptable reconstruction fidelity (MSE ≈0.00009). Values below 1.0 yielded latent features that were insufficiently discriminative (validation accuracy below 70%), while values above 3.0 degraded reconstruction quality without further improving classification performance. λ=3.0 thus represents the empirically identified point at which classification guidance dominates without collapsing the autoencoder’s ability to model the signal structure. This forced the encoder to learn latent features that are discriminative for stress classification rather than merely optimizing signal reconstruction. The resulting 52-dimensional feature vector (20 statistical + 32 latent) was globally normalized using z-score standardization (StandardScaler) so that no single feature dominated classifier behavior due to scale differences.This normalization was applied jointly to all 52 feature columns—handcrafted statistical and supervised autoencoder features alike—using a single StandardScaler fitted on the complete dataset, ensuring a consistent scale across both feature sources prior to classification. The hybrid feature set was then used as input for the classification algorithms, with the expectation that the combination of domain knowledge and data-driven learning would yield superior performance compared to either approach alone. An overview of the hybrid feature extraction architecture is shown in Figure 8, illustrating the parallel extraction of statistical and latent features and their subsequent fusion into a single feature vector for classification.

### 2.6. Classification and Model Training

Five classification algorithms were evaluated: (1) Decision Trees (DT); (2) Random Forest (RF)—an ensemble of multiple decision trees trained on random subsets of data and features; (3) K-Nearest Neighbors (KNN)—a distance-based algorithm using k=7 neighbors; (4) Support Vector Machines (SVM)—finding the optimal separating hyperplane with Platt scaling for probabilistic output; and (5) XGBoost—a sequential boosting algorithm. Additionally, ensemble combinations of RF and XGBoost using soft voting were evaluated. In soft voting, each base classifier *m* outputs a class-posterior probability vector ${\hat{p}}^{\left(m\right)}\left(c|x\right)$ for every class c∈{0,1} and input window x. The ensemble prediction is obtained by computing the weighted average of these probability vectors across all *M* classifiers,

(2) $${\hat{p}}_{\mathrm{ens}}\left(c|x\right)=\frac{\sum_{m=1}^{M}w_{m}\;{\hat{p}}^{\left(m\right)}\left(c|x\right)}{\sum_{m=1}^{M}w_{m}},$$

where $w_{m}\geq 0$ denotes the weight assigned to classifier *m*. The final class label is assigned to the class with the highest averaged probability: $\hat{y}=\arg\max_{c}\;{\hat{p}}_{\mathrm{ens}}\left(c|x\right)$. Two ensemble configurations were evaluated, both implemented via sklearn’s VotingClassifier with voting = ‘soft’. The first, Ensemble_RF_XGB, combined Random Forest and XGBoost with equal weights ($w_{\mathrm{RF}}=w_{\mathrm{XGB}}=1$). The second, Ensemble_All, combined four base classifiers—Random Forest (RF), XGBoost (XGB), Support Vector Machine (SVM), and K-Nearest Neighbors (KNN)—using asymmetric weights: $w_{\mathrm{RF}}=w_{\mathrm{XGB}}=2$ and $w_{\mathrm{SVM}}=w_{\mathrm{KNN}}=1$. The double weighting of RF and XGBoost reflects their consistently superior individual LOSO performance; SVM and KNN receive unit weight to contribute diversity without diluting the stronger classifiers. At each LOSO fold, all four base classifiers were trained independently on the training partition using their grid-search-optimized hyperparameters, and the ensemble prediction was formed by Equation (2). Unlike hard voting—which counts only the majority class label—soft voting exploits the full probability distribution and is therefore less susceptible to overconfident minority votes from individual classifiers. Unlike hard voting—which counts only the majority class label—soft voting exploits the full probability distribution and is therefore less susceptible to overconfident minority votes from individual classifiers.

To evaluate generalization to unseen individuals—a critical requirement for any wearable sensor system intended for deployment on new users—Leave-One-Subject-Out (LOSO) cross-validation was employed. In each of 15 iterations, data from one subject were held out entirely for testing, and the model was trained on the remaining 14 subjects. This strategy prevents data leakage and simulates real-world deployment, where a wearable sensor must provide accurate stress readings for a new, previously unseen wearer without prior calibration. To prevent any form of data leakage, all design choices involving training-data-dependent quantities—including the autoencoder loss weight λ, the latent dimensionality reduction from 64 to 32, and the classifier hyperparameters—were determined exclusively on training-fold data using inner cross-validation, and were fixed before evaluation on each held-out test subject. The z-score normalization parameters (mean and standard deviation per feature) were likewise computed on the training fold and applied to the test fold without refitting. Hyperparameter tuning was performed for all classifiers except Naive Bayes, which has no learnable hyperparameters, using exhaustive grid search (GridSearchCV) with a five-fold stratified inner cross-validation (StratifiedKFold, k=5) as the search criterion. Stratified splitting was applied to preserve the class ratio of approximately 1:3.4 (stress:non-stress) in each fold. The search was conducted on the complete dataset prior to LOSO evaluation, optimizing mean held-out accuracy across all inner folds. Table 4 lists the parameter grids explored for each classifier.

To prevent data leakage between the hyperparameter search and the subsequent LOSO evaluation, the best parameters identified by grid search were stored and used to instantiate fresh classifier objects within each LOSO fold; the refitted best_estimator_ from GridSearchCV was intentionally discarded. This ensures that no test-subject data could have influenced hyperparameter selection, preserving the integrity of the subject-independent evaluation.

## 3. Results

### 3.1. Preprocessing and Data Quality

Downsampling the raw 700 Hz RespiBAN chest-sensor EDA signal to 20 Hz reduced the total sample count from 31,470,603 to 875,420 (−97.2%) while retaining 97.4% of recording duration (50.0 min → 48.6 min), confirming that no physiologically relevant windows were lost to the resampling procedure. The anti-aliasing filter improved the mean SNR across subjects from 10.14dB to 18.22dB (+79.7%), demonstrating effective suppression of high-frequency noise. The movement ratio increased marginally from 0.044 to 0.060; residual motion-related contamination was addressed by the subsequent two-stage artifact detection procedure, which achieved a final artifact rate of 0.0% across all subjects. The two-stage artifact detection achieved a final artifact rate of 0.0% across all subjects and conditions, retaining 100% of time windows for analysis. Of 1841 initially flagged step artifacts, 1323 (71.9%) were reclassified as genuine SCR events through physiological validation, preventing substantial loss of true stress-related signal content. The adapted cvxEDA decomposition yielded 442 validated SCR peaks across 14 subjects; the stress condition consistently elicited the highest SCR rate (e.g., 12.2 peaks/min for Subject S2), confirming the physiological validity of the processing pipeline. The final merged dataset comprised 875,420 samples at 20 Hz, with a stress-to-non-stress ratio of approximately 1:3.4.

### 3.2. Feature Discrimination Across Extraction Methods

Statistical features extracted from 60-s windows (50% overlap, greater than 80% label purity, n=1374) showed clear physiological differentiation: the stress condition produced the highest mean maximum SCR amplitude (0.691±0.697μS), nearly double that of baseline (0.360±0.289μS), with the lowest relative variability (σ=9.2 for peak count vs. 18.1 for baseline). The unsupervised autoencoder achieved excellent reconstruction fidelity (MSE≈0.00009) and produced diverse, uncorrelated 64-dimensional latent features with clear arousal-based cluster separation in PCA space. However, LOSO classification using only these features reached only 54.2%, indicating that unsupervised reconstruction alone is insufficient for subject-independent stress detection from EDA sensor data.

### 3.3. Model Evaluation

The classification accuracy improved systematically through four architectural transitions, totaling a gain of 43.62 percentage points. The introduction of a weighted classification loss (λ=3.0) into the autoencoder training increased accuracy from 55% (unsupervised) to 89% (supervised), demonstrating that label-guided feature learning is essential. Fusion of 20 hand-crafted statistical features with 32 supervised latent features (hybrid approach) raised accuracy to 96.77%, confirming the complementarity of domain knowledge and deep learning. After hyperparameter optimization and the addition of XGBoost, all evaluated classifiers surpassed the PyEDA (v0.29.0) benchmark of 97.0%. PyEDA [17] is the open-source Python reference toolkit for EDA preprocessing and feature extraction, combining statistical descriptors with a convolutional autoencoder for automatic feature learning; evaluated on the wrist-based 4 Hz EDA channel of WESAD, it reports up to 97.0% binary stress classification accuracy and is widely used as the de-facto benchmark for single-modality EDA-based stress detection on this dataset. The final classification results for the hybrid feature set are shown in Table 5, demonstrating that all classifiers, including the simplest (Naive Bayes at 97.54%), exceeded the PyEDA benchmark, confirming that the discriminative power resides primarily in the feature representation rather than in classifier complexity.

The Ensemble RF + XGB achieved the highest mean accuracy of 98.00% (±1.76%) with an AUC of 0.994±0.008, attributed to the complementary strengths of Random Forest and XGBoost within the hybrid feature space. The consistent performance across all seven structurally distinct classifiers—from the parameter-free Naive Bayes (97.54%) to the best ensemble (98.00%)—suggests that the discriminative power resides primarily in the hybrid feature representation rather than in any individual model’s capacity to overfit the training data. Across all classifiers, Specificity exceeded 98% and AUC exceeded 0.985, confirming both low false-positive rates and strong ranking ability under class imbalance. Table 5 places these results in the broader context of published single-modality EDA stress detection approaches on WESAD. The proposed hybrid pipeline outperforms all listed baselines, including the most comparable recent work by Hosseini et al. [3,4] (97.03%) and PyEDA [17] (97.0%). It should be noted that a strict apples-to-apples comparison is limited by differences in sensor placement (wrist vs. chest EDA), sampling rate (4 Hz vs. 20 Hz), cross-validation protocol, and preprocessing strategy across studies.

### 3.4. Comparison of Feature Extraction Methods

**Figure 9:**
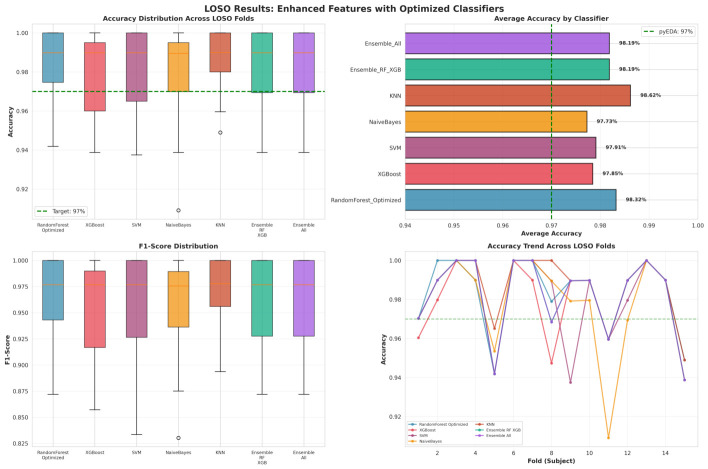
Classification accuracy across all classifiers and feature sets.

**Figure 10:**
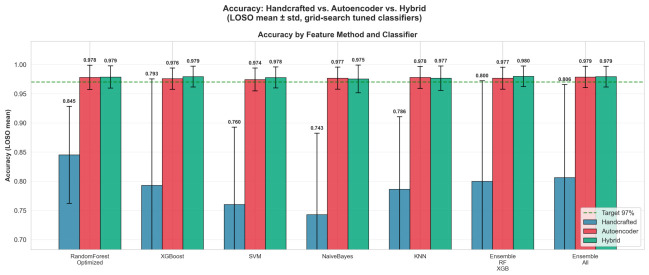
LOSO mean accuracy (± SD over 15 folds).

Table 6 summarizes the LOSO accuracy of the best-performing classifier (KNN) across all three feature extraction strategies evaluated in this study. The handcrafted feature set alone achieved 95.11%, confirming that physiologically grounded statistical descriptors—peak count, rise time, SCR amplitude, SCL slope—carry substantial discriminative information for binary stress classification. The supervised autoencoder features alone yielded 89.00%, a marked improvement over the unsupervised baseline (54.2%), but still below the handcrafted ceiling; this indicates that label-guided latent features capture complementary but not entirely overlapping information compared to the interpretable statistical features. The hybrid combination of both feature sets raised accuracy to 98.62%, outperforming both individual approaches by at least 3.51 percentage points and exceeding the PyEDA benchmark by 1.62 percentage points. This consistent superiority across all seven evaluated classifiers (see Figure 9 and Figure 10) confirms that the improvement is attributable to the complementarity of the two feature types rather than to any classifier-specific overfitting.

The performance gap between the handcrafted and autoencoder strategies illustrates their complementarity: statistical features encode well-understood physiological markers of sympathetic activation that generalize reliably across subjects, while the supervised autoencoder captures non-linear temporal dynamics that are difficult to define analytically. Neither source alone covers the full discriminative structure of the EDA signal; only their fusion produces the cluster geometry that enables near-perfect subject-independent classification.

### 3.5. Error Analysis

**Figure 11:**
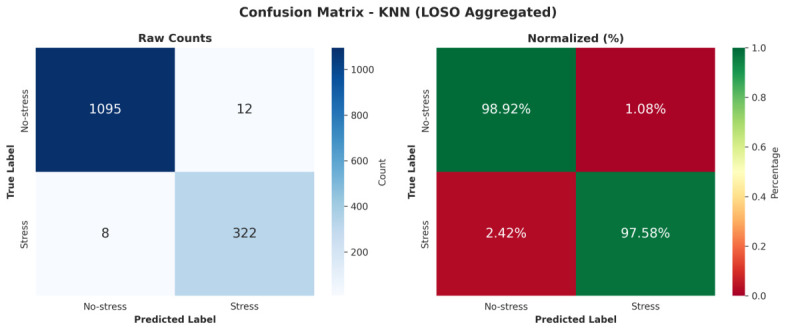
Confusion matrix for the best-performing KNN classifier.

The confusion matrix of the best-performing KNN model yielded a true positive rate (sensitivity) of 97.58% and a true negative rate (specificity) of 98.92%, limiting the false negative rate to 2.42% (Figure 11). In safety-critical applications such as firefighter monitoring, minimizing missed stress events is paramount; the low false negative rate represents a key strength of the proposed approach.

The 1.62 percentage point improvement over PyEDA corresponds to a 54% relative reduction in classification errors (from 3.0% to 1.38%), a substantial gain in a performance range where margins are increasingly difficult to improve.

## 4. Discussion

### 4.1. Significance of the Hybrid Feature Approach

The central finding of this study is that combining hand-crafted statistical features with label-guided autoencoder features produces classification performance that exceeds both individual approaches and the current state of the art on the WESAD dataset. The 43.62 percentage point progression from the unsupervised autoencoder (55%) to the final optimized hybrid model (98.62%) illustrates a key insight: reconstruction quality alone is a poor predictor of classification utility. The unsupervised autoencoder achieved near-perfect signal reconstruction (MSE≈0.00009) and produced diverse, uncorrelated latent features, yet these features proved largely indiscriminative for stress classification under LOSO evaluation. Only when the training objective was explicitly guided by stress labels (λ=3.0) did the latent features become discriminative, raising accuracy to 89%. The subsequent fusion with domain-specific statistical features (peak count, amplitude, rise time, SCL slope) contributed a further 7.77 percentage points, confirming that physiological domain knowledge and data-driven deep features capture complementary aspects of the EDA signal. Statistical features encode well-understood, interpretable markers of sympathetic activation, while the supervised autoencoder captures complex, non-linear temporal patterns that are difficult to define manually. The fact that all six evaluated classifiers exceeded the PyEDA benchmark of 97.0% when trained on the hybrid feature set, including the simplest model (Naive Bayes at 97.73%), demonstrates that the discriminative power resides primarily in the feature representation rather than in classifier complexity.

Placed in the broader context of the WESAD literature, the achieved 98.62% LOSO accuracy represents the highest reported single-modality EDA result to date. The original WESAD baseline reported 93.12% (binary, multi-modal LDA) and approximately 87.4% using chest EDA alone [5]. Subsequent reanalyses using conventional machine learning achieved comparable but not superior results: Bobade and Vani [18] reported up to 95.21% with a multi-layer perceptron on combined chest signals, and Gil-Martín et al. [19] obtained 96.62% with a convolutional neural network on a multi-modal feature stack. On wrist-based EDA alone, Hosseini et al. [3,4] reported 97.03% with AdaBoost and selected statistical features, while PyEDA [17] achieved 97.0% using a convolutional autoencoder. The 1.62 percentage point improvement of the proposed hybrid pipeline over PyEDA corresponds to a 54% relative reduction in classification errors (from 3.0% to 1.38%), demonstrating that the integration of physiologically informed handcrafted features with label-guided autoencoder features yields complementary benefits not captured by either approach in isolation.

### 4.2. Role of Sensor Technology and Signal Processing

The results underscore the critical role of sensor-specific signal processing in achieving high classification accuracy. The RespiBAN chest sensor’s 700 Hz sampling rate provided the temporal resolution necessary for rigorous anti-aliasing filtering prior to downsampling to 20 Hz, ensuring that no spectral artifacts were introduced during data reduction. The choice of chest-placed EDA over wrist-placed EDA (Empatica E4, 4 Hz) was validated by the extremely low final artifact rate (0.0%) and the high SCR preservation rate (71.9% of initially flagged artifacts reclassified as genuine physiological responses). These results suggest that for laboratory and controlled-environment studies, chest-sensor EDA provides sufficient signal quality to support single-modality stress detection without the need for multi-sensor fusion. The adaptive threshold approach for artifact detection (Factor×MAD) proved essential for accommodating the substantial inter-subject variability inherent in EDA sensor readings, with step thresholds ranging from 1.255 to 6.548 across subjects. A fixed-threshold approach would have either missed artifacts in high-variability subjects or falsely flagged genuine SCR events in low-variability subjects. Similarly, the cvxEDA parameter adaptations—particularly the reduction of the sparsity penalty α from $8\times 10^{-4}$ to $4\times 10^{-4}$—were necessary to prevent over-smoothing of the phasic SCR component at the reduced 20 Hz sampling rate. These findings highlight that standard signal processing parameters developed for high-frequency laboratory amplifiers cannot be applied directly to downsampled or lower-resolution wearable sensor data without careful recalibration.

### 4.3. Subject-Independent Generalization

The use of strict LOSO cross-validation ensures that all reported metrics reflect generalization to entirely unseen individuals, simulating real-world deployment where a wearable sensor must provide accurate stress readings for a new user without prior calibration. The KNN classifier exhibited the smallest inter-fold variance across the 15 LOSO iterations, indicating robust performance despite the considerable inter-subject variability in EDA baseline levels, SCR amplitude ranges, and autonomic reactivity. This stability is particularly relevant for the intended application in firefighter monitoring, where the system must function reliably across a diverse user population with varying physiological profiles. The false negative rate of 2.42% is especially noteworthy in this application context. In safety-critical environments, failing to detect a stressed state can impair decision-making and endanger both the responder and the individuals they protect. The high sensitivity (97.58%) achieved without sacrificing specificity (98.92%) indicates that the hybrid approach effectively balances the competing risks of missed alarms and false alarms.

### 4.4. Computational Feasibility and Deployment Considerations

**Figure 12:**
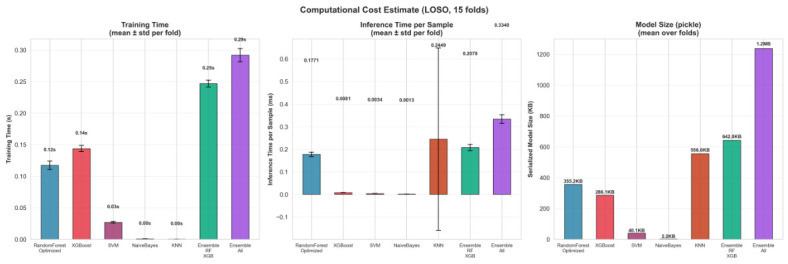
Computational cost estimate across all classifiers (LOSO, 15 folds). **Left**: mean training time per fold (±SD). **Centre**: mean per-sample inference latency (±SD). **Right**: mean serialised model size (pickle). All measurements recorded on an Intel Core i7-12700H, 32 GB RAM, no GPU acceleration.

Figure 12 summarises the computational cost of all evaluated classifiers across three deployment-relevant dimensions: training time, per-sample inference latency, and serialised model size, each averaged over 15 LOSO folds.

Training is performed offline and therefore imposes no real-time constraint; all classifiers complete a training fold in under 0.30 s on standard hardware. Per-sample inference latency ranges from 0.001 ms (Naive Bayes) to 0.334 ms (Ensemble All). At the sensor’s operating rate of 20 Hz, a new EDA sample arrives every 50 ms, meaning that even the slowest ensemble consumes less than 0.7% of the available inter-sample budget. Notably, KNN exhibits substantially higher inter-fold variance in inference latency (mean 0.245 ms, SD visible in Figure 12) than all other classifiers. This is expected: unlike parametric models, KNN performs no explicit model compression during training and instead searches the full retained training set at inference time, making its latency directly proportional to the number of training windows, which varies across LOSO folds as subject sizes differ. Serialised model sizes range from 2.2 KB (Naive Bayes) to 1.2 MB (Ensemble All).

These figures indicate a clear deployment spectrum across three hardware tiers that are representative of operational first-responder equipment:

Tier 1—On-device (microcontroller-class wearable). Naive Bayes (2.2 KB, 0.001 ms) and SVM (40.1 KB, 0.003 ms) fit comfortably within the flash and SRAM constraints of common wearable system-on-chips such as the Nordic nRF52840 (1 MB flash, 256 KB SRAM) or the STM32L4 series. These classifiers can execute stress detection entirely on the sensor node without offloading, making them suitable for scenarios where continuous wireless transmission is infeasible due to power or bandwidth constraints.

Tier 2—On-device (higher-end embedded SoC). Random Forest (355 KB, 0.177 ms) and XGBoost (286 KB, 0.008 ms) are compatible with platforms such as the Nordic nRF5340 (1 MB flash, 512 KB SRAM) or the ESP32-S3, both of which appear in current professional-grade wearable body-area network nodes. XGBoost in particular combines near-RF accuracy (97.93%) with a smaller footprint and lower inference latency, making it the pragmatically optimal single-model choice for embedded deployment.

Tier 3—Edge-computing companion. The ensemble variants (556 KB–1.2 MB) exceed the RAM envelope of standalone wearable MCUs but remain well within the capabilities of a Raspberry Pi Zero 2 W (512 MB LPDDR2) or equivalent low-power companion processor that could be integrated into a chest harness or belt unit alongside the sensor node. Given that the Ensemble RF + XGB achieves the highest accuracy (98.00%) with an inference latency of only 0.208 ms, this tier represents the best accuracy–cost trade-off for applications where a companion chip is acceptable.

It should be noted that the present measurements were obtained on a desktop-class CPU (Intel Core i7-12700H, 32 GB RAM) and represent an upper bound on achievable speed rather than a direct embedded benchmark. Porting to a bare-metal MCU environment would require quantisation or a C-compiled decision-tree representation (e.g., via micromlgen or emlearn), which typically reduces both model size and latency by an additional factor of two to five. A dedicated embedded validation is planned as future work.

### 4.5. Limitations

Several limitations should be considered when interpreting these results. First, the WESAD dataset was collected in a controlled laboratory setting, where common field-deployment challenges—such as extreme motion artifacts, high ambient temperatures, and prolonged recording durations—were minimized. The 0.0% artifact rate observed in this study is unlikely to be replicated in real-world firefighting scenarios, where heavy physical exertion and extreme heat will significantly increase sensor noise and may elevate tonic skin conductance independently of psychological stress. Second, the study relied exclusively on chest-placed EDA. While this placement yielded superior signal quality, it is less practical for long-term field deployment than wrist-worn sensors. Translating the pipeline to wrist-sensor data (e.g., Empatica E4 at 4 Hz) would require substantial adaptation of the filtering, artifact detection, and cvxEDA parameters, and would likely encounter higher artifact loads due to hand and arm movements. Nevertheless, the WESAD dataset includes a synchronised wrist-based EDA channel (Empatica E4, 4 Hz), which provides a direct basis for evaluating the proposed hybrid feature extraction under wrist-sensor conditions without additional data collection. Hosseini et al. [3,4] demonstrated that carefully selected statistical features from the same wrist channel already achieve 97.03% accuracy, suggesting that the discriminative signal is present in wrist EDA and that the addition of supervised autoencoder features has the potential to yield further improvements. Adapting the pipeline to the wrist channel is therefore a concrete and low-barrier next step, requiring primarily a recalibration of downsampling, artifact thresholds, and the cvxEDA sparsity parameter α to account for the lower sampling rate and higher motion susceptibility of the wrist placement. Finally, the sample size of 15 subjects (14 after exclusion of S12), while standard for the WESAD benchmark, limits the statistical power of cross-subject generalization claims. Replication on larger cohorts is necessary to confirm the robustness of the hybrid approach across broader demographic and physiological variation.

### 4.6. Future Directions

The most immediate extension is the direct evaluation of the hybrid pipeline on the wrist-based EDA channel already present in the WESAD dataset (Empatica E4, 4 Hz). This would quantify the accuracy trade-off between chest and wrist placement under identical experimental conditions and feature extraction logic, providing a principled answer to the practical deployment question. The subsequent step is the integration of a co-located three-axis accelerometer within a single wristband to enable motion artifact rejection without requiring a chest strap. Both EDA and accelerometer sensors are compact and low-power, making this the most practical dual-sensor configuration for field use. The accelerometer signal would allow the system to distinguish EDA increases caused by physical movement from those caused by genuine psychological stress, addressing the primary limitation of single-modality EDA.

A second extension concerns generalization beyond the WESAD dataset. The controlled laboratory protocol of WESAD—based on the Trier Social Stress Test with 15 subjects—captures only a narrow slice of the stressor space encountered in real-world deployment. Validation on additional public datasets that vary in stressor type, sensor platform, and label structure is therefore essential. Three complementary datasets are particularly relevant: SWELL-KW [20], which captures office-work stressors (e.g., interruptions, time pressure) using physiological and behavioural modalities and is the closest analogue to chronic occupational stress; DriveDB [21], which provides EDA recordings from real-world driving tasks at varying traffic-load levels and represents a high-motion field condition relevant to the firefighter use case; and StressID [22], which uniquely offers continuous numerical self-reports of perceived stress, arousal, and valence, enabling direct regression-based stress intensity prediction rather than derivation of a stress score from binary classification probabilities. For broader affective generalization, DEAP [23] provides EDA recordings in a high-arousal video-elicitation paradigm covering both stress and non-stress affective states. Cross-dataset evaluation across these benchmarks would clarify whether the hybrid feature representation captures sensor- and stressor-invariant components of the sympathetic response or whether recalibration of the supervised autoencoder per dataset is required.

Finally, the ultimate test of the proposed approach is real-world deployment in a firefighter cohort. This would involve collecting EDA data from firefighters during training exercises and actual deployments, applying the developed pipeline to detect stress events, and correlating these detections with self-reports, performance metrics, and physiological outcomes (e.g., heart rate variability) to validate the system’s real-world utility and safety implications.

## 5. Conclusions

This study presented a complete processing pipeline for the detection and quantification of psychological stress from chest-worn EDA sensor data, evaluated on the publicly available WESAD dataset with firefighters as the target application. The pipeline encompasses signal conditioning (filtering, downsampling from 700 Hz to 20 Hz), a physiologically informed two-stage artifact detection procedure, adapted cvxEDA decomposition, and a hybrid feature extraction method that fuses 20 hand-crafted statistical features with 32 label-guided autoencoder features. The optimized KNN classifier achieved a peak accuracy of 98.62% under strict LOSO cross-validation, surpassing the previous state of the art (PyEDA, 97.0%) by 1.62 percentage points and reducing the classification error rate by 54%. The model attained a sensitivity of 97.58% and a specificity of 98.92%, limiting the false negative rate to 2.42%—a critical metric for safety-critical applications where missed stress events pose a direct risk to personnel. Three principal conclusions emerge from this work. First, label-guided feature learning is essential for EDA-based stress detection. Unsupervised autoencoder features, despite excellent reconstruction fidelity, yielded only 54.2% LOSO accuracy, whereas the introduction of a weighted classification loss raised accuracy to 89% and the subsequent hybrid fusion to 98.62%. Second, careful adaptation of signal processing parameters to the specific sensor platform and sampling rate is indispensable; standard cvxEDA and artifact detection parameters designed for high-frequency laboratory data cannot be transferred to downsampled wearable sensor signals without recalibration. Third, a single EDA sensor—when combined with rigorous preprocessing and hybrid machine learning—can achieve classification performance comparable to multi-modal approaches, supporting the feasibility of lightweight, non-invasive wearable stress monitoring for high-risk occupations. While the present results are based on chest-worn EDA, the availability of a synchronised wrist-based EDA channel in WESAD provides a direct pathway for evaluating the pipeline’s transferability to the wrist placement that dominates consumer wearable devices, and this comparison is identified as the most immediate direction for future work.

## Figures and Tables

**Table 1:** Dataset metrics before and after downsampling, aggregated across all 14 subjects (means).

Metric	Before (700 Hz)	After (20 Hz)	Δ
Total Samples	31,470,603	875,420	−97.2%
Duration (min)	50.0	48.6	−2.6%
SNR (dB)	10.14	18.22	+79.7%
Movement Ratio	0.044	0.060	+35.1%

**Table 2:** Comparison of cvxEDA parameters: original vs. adapted for the downsampled RespiBAN signal.

Parameter	Original (Lab Data)	Adapted (WESAD 20 Hz)
Sampling Rate	250–1000 Hz	20 Hz
$\tau_{0}$	2.5 s	2.0 s
$\tau_{1}$	0.7 s	1.0 s
α	$8\times 10^{-4}$	$4\times 10^{-4}$
$\delta_{\mathrm{knot}}$	10 s	20 s
Peak Threshold	0.05μS (fixed)	Adaptive (condition-aware)

**Table 3:** Overview of the 20 hand-crafted features extracted per 60-s window.

Group	Feature	Description	Unit
SCR	scr_mean	Mean amplitude of the phasic component	μS
	scr_std	Standard deviation of SCR	μS
	scr_max	Maximum SCR value within window	μS
	scr_min	Minimum SCR value within window	μS
	scr_peak_count	Number of peaks exceeding 0.05 μS threshold	—
	scr_peak_mean_amp	Mean amplitude of all detected peaks	μS
	scr_peak_max_amp	Maximum amplitude among detected peaks	μS
	scr_peaks_per_min	SCR peak occurrence rate	min${-}^{1}$
	scr_avg_rise_time	Mean onset-to-peak rise time of detected peaks	s
	scr_auc	Area under positive SCR curve (trapezoidal integration)	μS·s
SCL	scl_mean	Mean tonic skin conductance level	μS
	scl_std	Standard deviation of SCL	μS
	scl_max	Maximum SCL value within window	μS
	scl_min	Minimum SCL value within window	μS
	scl_slope	Linear regression slope of SCL (arousal trend)	μS/sample
	scl_range	Dynamic range (max−min)	μS
	scl_variation	Standard deviation of first differences (rate of change)	μS
Quality	signal_snr	Mean signal-to-noise ratio of the window	dB
	movement_ratio	Fraction of samples affected by movement artifacts	—
Spectral	eda_spectral_power_low	Welch PSD power in the 0–0.5 Hz stress-related band	μS^2^/Hz

**Table 4:** Hyperparameter search grids used in GridSearchCV (k=5 stratified inner CV, scoring: accuracy).

Classifier	Parameter	Values Searched
Random Forest	n_estimators	100, 200, 300
	max_depth	10, 15, 20, *None*
	min_samples_split	2, 5
	min_samples_leaf	1, 2
	max_features	*sqrt*, *log2*
XGBoost	n_estimators	100, 200, 300
	max_depth	4, 6, 8
	learning_rate	0.01, 0.05, 0.10
	subsample	0.7, 0.8, 1.0
	colsample_bytree	0.7, 0.8, 1.0
	gamma	0, 0.1, 0.3
SVM	C	0.1, 1, 10, 100
	kernel	*rbf*, *poly*
	gamma	*scale*, *auto*
KNN	n_neighbors	3, 5, 7, 9, 11
	weights	*uniform*, *distance*
	metric	*euclidean*, *manhattan*

**Table 5:** Final classification results with hybrid features (LOSO cross-validation, mean ± SD over 15 folds) and comparison with related work on the WESAD dataset. Direct numerical comparisons are constrained by differences in cross-validation protocols, sensor placement, and preprocessing strategies. Acc. = Accuracy; Sens. = Sensitivity; Spec. = Specificity.

Method	Sensor	Acc. (%)	F1	Sens.	Spec.	AUC	Δ PyEDA
Related work on WESAD (binary stress classification)							
Zhu et al. [2]	Wrist	86.50	—	—	—	—	−10.50%
Schmidt et al. [5]	Chest	87.40	—	—	—	—	−9.60%
Hosseini et al. [3,4]	Wrist	97.03	—	—	—	—	−0.03%
PyEDA [17]	Wrist	97.00	—	—	—	—	—
This work (chest EDA, hybrid features, mean ± SD over 15 LOSO folds)							
Ens. RF + XGB	Chest	98.00±1.76	0.957±0.037	0.952±0.058	0.989±0.020	0.994±0.008	+1.00%
XGBoost	Chest	97.93±1.78	0.955±0.038	0.952±0.064	0.988±0.020	0.993±0.009	+0.93%
Ens. All	Chest	97.93±1.78	0.955±0.037	0.949±0.062	0.989±0.020	0.993±0.012	+0.93%
RF (opt.)	Chest	97.87±1.90	0.954±0.039	0.949±0.062	0.988±0.023	0.993±0.009	+0.87%
SVM	Chest	97.79±1.81	0.950±0.039	0.928±0.066	0.993±0.016	0.988±0.016	+0.79%
KNN (k=7)	Chest	97.66±2.10	0.949±0.044	0.944±0.071	0.987±0.024	0.985±0.018	+0.66%
Naive Bayes	Chest	97.54±2.36	0.949±0.044	0.962±0.051	0.980±0.032	0.988±0.012	+0.54%

**Table 6:** LOSO accuracy of the best classifier (KNN) per feature extraction method.

Feature Method	Features	KNN Accuracy	Δ vs. Hybrid
Handcrafted (statistical only)	20	95.11%	−3.51 pp
Autoencoder (supervised, latent only)	32	89.00%	−9.62 pp
**Hybrid (handcrafted + autoencoder)**	**52**	**98.62%**	**—**
PyEDA benchmark [17]	—	97.00%	−1.62 pp

## Data Availability

The data presented in this study are available on request from the corresponding author due to (specify the reason for the restriction).

## Abbreviations

The following abbreviations are used in this manuscript:

EDA	Electrodermal Activity
SCR	Skin Conductance Response
SCL	Skin Conductance Level
SNS	Sympathetic Nervous System
LOSO	Leave-One-Subject-Out
KNN	K-Nearest Neighbors
SVM	Support Vector Machine
RF	Random Forest
MSE	Mean Squared Error
MAD	Median Absolute Deviation
WESAD	Wearable Stress and Affect Detection
TSST	Trier Social Stress Test
